# Strengths of primary healthcare regarding care provided for chronic kidney disease**^1^**

**DOI:** 10.1590/1518-8345.1234.2801

**Published:** 2016-09-09

**Authors:** Elaine Amaral de Paula, Mônica Barros Costa, Fernando Antonio Basile Colugnati, Rita Maria Rodrigues Bastos, Chislene Pereira Vanelli, Christiane Chaves Augusto Leite, Márcio Santos Caminhas, Rogério Baumgratz de Paula

**Affiliations:** 2 MSc, RN, Hospital Escola da Universidade Federal de Pelotas, Pelotas, RS, Brazil.; 3 PhD, Full Professor, Faculdade de Medicina, Universidade Federal de Juiz de Fora, Juiz de Fora, MG, Brazil.; 4 PhD, Adjunct Professor, Faculdade de Medicina, Universidade Federal de Juiz de Fora, Juiz de Fora, MG, Brazil.; 5 PhD, Physician, Secretaria Municipal de Saúde, Juiz de Fora, MG, Brazil.; 6 Doctoral student, Universidade Federal de Juiz de Fora, Juiz de Fora, MG, Brazil.; 7 MSc, Physician.; 8 Physician.

**Keywords:** Process Assessment (Health Care), Outcome Assessment (Health Care), Delivery of Health Care, Chronic Kidney Disease

## Abstract

**Objective:**

to assess the structure and results obtained by the "Chronic Renal Patients Care Program" in a Brazilian city.

**Method:**

epidemiological, cross-sectional study conducted in 14 PHC units and a secondary center from 2010 to 2013. The Donabedian Model was the methodological framework used. A total of 14 physicians, 13 supervisors, and 11 community health agents from primary healthcare were interviewed for the assessment of structure and process and 1,534 medical files from primary healthcare and 282 from secondary care were consulted to assess outcomes.

**Results:**

most units lacked sufficient offices for physicians and nurses to provide consultations, had incomplete staffing, and most professionals had not received proper qualification to provide care for chronic renal disease. Physicians from PHC units classified as capable more frequently referred patients to the secondary care service in the early stages of chronic renal disease (stage 3B) when compared to physicians of units considered not capable (58% vs. 36%) (p=0.049). Capable PHC units also more frequently presented stabilized glomerular filtration rates (51%) when compared to partially capable units (36%) and not capable units (44%) (p=0.046).

**Conclusion:**

patients cared for by primary healthcare units that scored higher in structure and process criteria presented better clinical outcomes.

**Objective:**

to identify the coping strategies of family members of patients with mental disorders and relate them to family member sociodemographic variables and to the patient's clinical variables.

**Method:**

this was a descriptive study conducted at a psychiatric hospital in the interior of the state of São Paulo, with 40 family members of hospitalized patients over the age of 18, and who followed the patient before and during hospitalization. We used tools to characterize the subjects and the Folkman and Lazarus Inventory of Coping Strategies.

**Results:**

the coping strategies most often used by family members were social support and problem solving. Mothers and fathers used more functional strategies (self-control p=0.037, positive reappraisal p=0.037, and social support p=0,021). We found no significant differences between the strategies and other variables examined.

**Conclusion:**

despite the suffering resulting from the illness of a dear one, family members make more use of functional strategies, allowing them to cope with adversities in a more well-adjusted way.

## Introduction

The Brazilian health system is experiencing a situation characterized by high rates of morbidity and mortality caused by chronic conditions, among which the following stand out: type 2 diabetes mellitus, hypertension, chronic kidney disease (CKD), and cardiovascular diseases^1^.

Concomitantly, infectious diseases persist, along with the strong growth of external causes, which combine to form a complex epidemiological situation defined as "triple burden of disease"^2^. 

Changes in the population's epidemiological profile are not properly heeded by the organization of the healthcare system so that inconsistency between the population's needs and the current healthcare model is observed^2^. Hence, coping with chronic conditions is a challenge faced by managers and professionals in all the fields of the healthcare system, as well as by patients. 

Specifically in the case of CKD, severe problems still persist due to a low level of problem-solving capacity in primary healthcare (PHC)^3^ ^-^ ^4^. Studies show that this disease is highly prevalent and mainly affects elderly individuals, hypertensive and diabetic patients, and is often under-diagnosed, untreated, or addressed only later on^5^ ^-^ ^6^.

Data concerning the world population reveals that the prevalence of CKD is between 8% and 16%^6^. To date, there is no definitive information concerning the epidemiology of CKD in Brazil. Data obtained by a clinical laboratory in Juiz de Fora, MG, Brazil from 24,248 adult individuals when the disease was diagnosed using glomerular filtration rate (GFR) taking two creatinine blood test with a minimum interval of three months, revealed that 2.3% of the individuals presented CKD stages 3B, 4 or 5. If this percentage is extrapolated to the Brazilian adult population, an estimate of thee million individuals with CKD in advanced stage is obtained^7^. The high prevalence of this disease associated with the complexity of its treatment results in the consumption of 10% of the annual budget of the Brazilian Ministry of Health, in addition to loss of productivity and of quality of life^8^.

Given the need to design new proposals to guide prevention strategies and the management of chronic diseases, the Ministry of Health has developed policies to reorganize the health services focusing on Integrated Health Services Networks. In Minas Gerais, this proposal was consolidated with the creation of the HIPERDIA Minas Program, characterized by the supply of care shared between PHC units and Hiperdia Centers^9^.

In this model, the PHC is the entry way to the health system and should monitor the entire path the patient goes through the network by using specific tools such as: clinical records, guidelines, a referral and counter-referral system, and effective communication strategies between PHC teams and specialists. 

Understanding the complexity of care provided to patients with CKD and the role of PHC in the coordination of the Integrated Health Services Networks, this study's objective was to assess the structure, process, and results obtained by the Chronic Renal Patients Care Program in a Brazilian city.

## Method

This was a cross-sectional epidemiological study conducted in 14 PHC units and a secondary healthcare center enrolled in the Hiperdia Minas Program in Juiz de Fora, MG, Brazil between 2010 and 2013.

The Donabedian Model^10^ was used to construct the structure, process and results indicators. 

The variables related to structure include the qualification of human resources and the availability of physical and material resources in the units. The process variables refer to screening actions of risk factors for CKD, criteria used to diagnose this disease and to refer patients to the Hiperdia Minas Program. Results were assessed based on clinical indicators, such as: average of the last two blood pressure measurements; fasting glucose; and serum creatinine recorded in the medical files of individuals monitored by the hypertension and diabetes mellitus groups from the units under study. 

The technical and normative guidelines established for the Hiperdia Minas Program were considered in this study. According to the State Health Department's Resolution No. 2,606/2010, patients with hypertension and/or diabetes mellitus presenting the following conditions must be referred to the chronic kidney disease ambulatory at the Hiperdia Minas Center in Juiz de Fora: - CKD stage 3B, 4 or 5; estimated annual loss of glomerular filtration ≥5 mL/min/year (initial GFR - final GFR/number of months of observation x 12); - proteinuria>1.0 g/day or proteinuria<1.0 g/day accompanied by hematuria; - abrupt increase of serum creatinine (≥30%); 25% decrease in estimated GFR when initiating some medication that blocks the renin-angiotensin- aldosterone axis. These guidelines were provided to the PHC units at the time the program was implemented in the city.

The PHC units were selected according to region criteria and the frequency with which patients were referred to the secondary health care service. Hence, two units of each of the seven administrative regions in the city (north, south, east, west, northeast, southeast and center) were selected: the one with the highest number of referrals and another with the lowest number of referrals to the Hiperdia Minas Center in Juiz de Fora. The professionals were randomly selected for the interviews. The PHC units selected for this study represented 23% of all units in the city, covering approximately 63,000 users of the healthcare system.

The instrument used^11^ was originally designed for a normative assessment of the care provided to individuals with diabetes mellitus. This instrument was adapted for this study to allow the care provided to patients with CKD to be assessed. Indicators and actions were selected according to the "Guidelines for Care Provided to Chronic Kidney Disease"^12^.

To analyze the capability of PHC units, scores were assigned to each criterion of the questionnaire and these scores were totaled within the structure or process' subcomponents. This way, we have two columns: one presenting the sum of the structure's subcomponents and another presenting the sum of the process's subcomponents for each interview. The structure dimension received a weight of 4 and the process dimension received a weight of 6. The scores obtained by the three interviewees in the subcomponents of both structure and process were totaled to reach a PHC unit's total score, which in turn represents a unit's capability. This score was then divided into terciles of classification and, based on the total scores, each PHC unit was classified as "capable", "partially capable", or "not capable", that is, these classifications concern the PHC units' capacity to implement preventive actions and monitor CKD. 

A total of 1,534 medical files from PHC that corresponded to the micro-area of the physician interviewed were selected to be analyzed. These files contained the clinical information of the patients cared for by the hypertension and/or diabetes mellitus groups, beginning in 2010. Another 282 files of patients with CKD who were referred to the Hiperdia Center by the PHC units selected for the study in the period in which the program was implemented (from September 2010 to August 2013) were also selected. 

According to the progression of the GFR over the follow-up period, patients were classified as: - non-progressing (GFR did not decrease); - slowly progressing (GFR decreased up to 5 mL/min/year); - rapidly progressing (GFR decreased ≥5 mL/min/year). Blood pressure was considered to be under control when systolic blood pressure was ≤ 140 mmHg and/or diastolic blood pressure was ≤ 90 mmHg among hypertensive individuals. Those with diabetes should have their systolic blood pressure ≤ 130 mmHg and/or diastolic blood pressure ≤ 80 mmHg^13^.

The variables selected were pre-encoded and stored in SPSS^(r)^ *(Statistical Package for the Social Sciences* ) version 15.0. Data were interpreted using descriptive analysis, such as means and prevalence. The relationships among variables were assessed with Chi-square test. A level of significance of 5% and confidence interval of 95% were adopted for all statistics. The study was approved by the Institutional Review Board at the university hospital of Juiz de Fora (report No. 133.399). The participants signed free and informed consent forms.

## Results

Among the 14 PHC units selected, 11 were Family Health Strategy (FHS) units, two were traditional PHC units, and one was mixed (FHS and traditional PHC). A total of 14 physicians, 13 supervisors, and 11 community health agents were interviewed.

The average time the professionals worked in the PHC unit was 6.0±6.12 years, 8.3±6.31 and 8.6±3.55 years, for physicians, supervisors and community health agents, respectively; 57% of the physicians, 39% of the supervisors and 23% of the community health agents had worked less than five years in the unit.

In regard to the physical structure, nine (61%) units did not have a sufficient number of offices for physicians and nurses to simultaneously give consultations, while ten (69%) units did not have sufficient amounts of basic medication to meet the demand they experienced.

Staffing for only four (31%) of the 14 PHC units was complete. In regard to qualification, eight physicians (54%) and 11 nurses (77%) had specialization in Family Health and seven (67%) community health agents had attended the introductory course in Family Health. Additionally, only eight (54%) physicians, six (46%) nurses and one community health agent had received specific qualification to provide care and assist patients with CKD.

None of the 14 PHC units had guidelines specific for CKD. The interviews with the physicians revealed that six (43%) of them knew the criteria used to refer patients to the Hiperdia Minas Center and nine physicians (64%) used serum creatinine as the main criterion to refer patients to a secondary care service. Additionally, only five (36%) interviewees reported including urinary protein excretion as an assessment criterion. These figures are even lower when the referral criteria, that is, checking for glomerular filtration rate and microalbuminuria screening (which are used to establish how severe the disease is), are considered. Hence, less than one third of the physicians reported using the GFR and only one mentioned that microalbuminuria was an assessment criteria. Two participants reported using GFR provided by the clinical analysis laboratory. 

In regard to the coordination of the flow of patients within the network, the interview with the professionals from the PHC units revealed there is no committee to regulate referrals to the specialist in the city. 

Therefore, considering the PHC units' structure and process aspects, their capacity to prevent risk factors, and early detection and control of CKD, was classified as followed: five (36%) units were classified as capable, five (36%) as partially capable, and four (28%) as not capable.

When analyzing the 1,534 medical files of patients monitored by the hypertension and diabetes mellitus groups from the PHC units, we observed errors in the recording of routine laboratory exams. In four years of follow-up, no data regarding fasting glucose were recorded in 18% (276) of the medical files; 163 (44%) files of diabetic patients presented no records of glycated hemoglobin; and no records of serum creatinine, which refers to screening for CKD, were found in 398 (26%) files, while only one record was found in another 261 (17%) files.

CKD was diagnosed in 250 (16%) individuals based on the GFR. Table presents the description of the sample's sociodemographic and clinical characteristics.

**Table 1:** Socidemographic and clinical characterization of chronic renal patients cared for in PHC units in the city of Juiz de Fora, MG, Brazil, 2010 to 2013

Variables		Total (N=250)
Average age, years (Standard deviation)		67 (10.9)
Men, n(%)		204 (82)
Comorbidities		
	Hypertension, n(%)	175 (70)
	Diabetes *mellitus*, n(%)	75 (30)
Chronic kidney disease staging		
	Stage 3 A	143 (57)
	Stage 3B	88 (35)
	Stage 4	15 (6)
	Stage 5	4 (2)

The analysis of clinical indicators showed that 61% of the patients cared for by the capable PHC units had their average blood pressure under control, while only 39% of those cared for by PHC units were classified either as partially or not capable had their average blood pressure under control (p=0.92).

**Figure 1:**
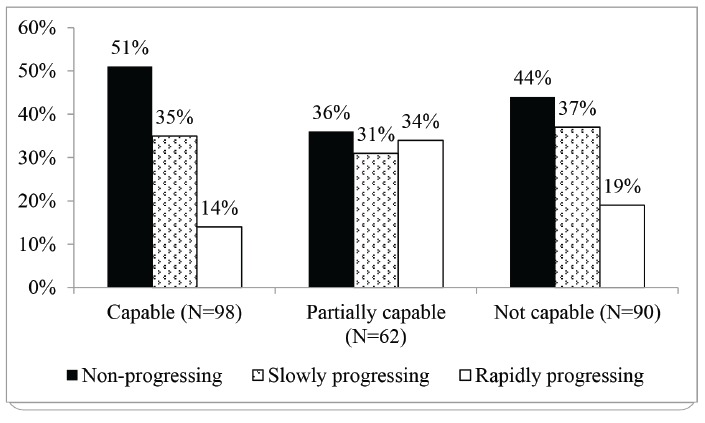
Progression of the GFR of patients monitored by PHC unit, according to their classification. Juiz de Fora, Minas Gerais, Brazil, 2010 to 2013 (N=250)

In regard to the GFR progression, 51% of the patients monitored in the capable PHC units had stabilized their GFR, while 56% of those monitored in PHC units that were not capable presented slow or rapid decline of the GFR (p=0.046) (Figure 1).

**Figure 2:**
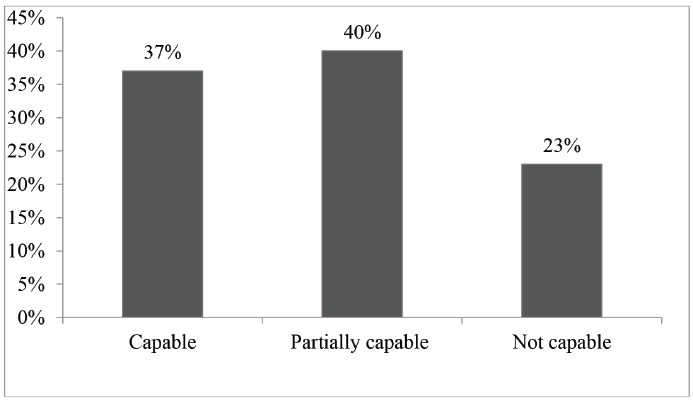
Frequency in which patients were referred to the Hiperdia Minas Center in Juiz de Fora, according to the PHC unit's classification. Juiz de Fora, Minas Gerais, Brazil, 2010 to 2013 (N=282)

The analysis of the conditions under which patients were referred to the Hiperdia Minas Center's chronic kidney disease ambulatory unit in Juiz revealed that the "not capable" PHC units less frequently referred patients to the ambulatory unit compared to "capable" and "partially capable" units (p<0.001) (Figure 2).

The PHC units classified as "capable" more frequently presented knowledge regarding the criteria used to refer patients to secondary services (80%), compared to units considered partially capable (40%) or not capable (50%).

**Figure 3:**
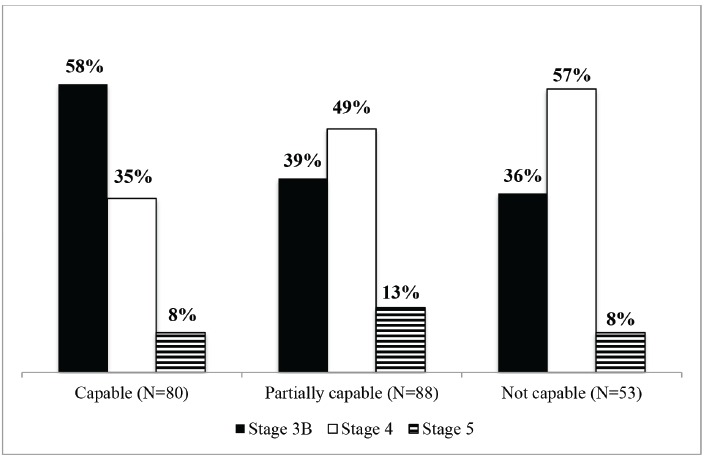
Stage of the chronic kidney disease among patients referred to the Hiperdia Minas Center in Juiz de Fora, according to the PHC unit classification. Juiz de Fora, MG, Brazil, 2010 to 2013 (N = 221).

When assessing the stages of the CKD at the time of referral, 61 (22%) patients were at stage 3A, 99 (35%) were at stage 3B, 101 patients (36%) were at stage 4, and 21 (7%) were at stage 5. Considering that being at stage 3B is a criterion to refer a patient to a secondary care service, the analysis of the patients' stages at the time of referral according to the PHC units' classifications revealed that 58% of patients originating from units considered capable were at stage 3B. In turn, those units classified as not capable had referred most of their patients (65%) when they were already at stage 4 or 5 of CKD (p=0.049) (Figure 3).

## Discussion

This study's results reveal the weaknesses and strengths presented by the healthcare network available to chronic renal patients in Juiz de Fora, MG, Brazil. If, on the one hand, assessment of the capacity of PHC units to provide care to individuals with CKD showed a lack of resources and systematization of preventive and monitoring actions to manage kidney disease, on the other hand, strengths such as improved results were observed in the care provided to individuals with chronic conditions in PHC units that have more well-established structure and processes. 

A deficient physical structure and a lack of material resources in the units were also observed. Most units had an insufficient number of offices for physicians and nurses to concomitantly provide consultations and insufficient medication to meet the demands of patients. 

We also observed that most professionals were not specifically qualified to provide care to patients with CKD. In regard to this aspect, the obstacle most frequently mentioned by the units' managers was the low number of workers with the appropriate profile and technical qualification to enable the team's expansion process. Other factors, such as lack of social recognition, difficulties attending continuing education programs, poor working conditions, and difficulties faced in the management of the staff, also contribute to high turnover and consequent fragmentation of the network^14^ ^-^ ^15^.

In addition to the structural and human resources deficiencies, weaknesses regarding the process were also observed. Thus, a lack of guidelines concerning CKD and a lack of knowledge concerning criteria used to refer patients to secondary care services observed among the units under study possibly resulted in delayed diagnoses and referrals. As seen in the interviews with the PHC physicians, serum creatinine was the main parameter used to assess renal function, at the expense of the GFR. Additionally, most physicians failed to mention microalbuminuria as a criterion to be considered in the referral of patients to specialized care. Similarly, analysis of the medical files of hypertensive and diabetic patients cared for in the PHC units revealed that only 57% of the files contained two creatinine measurements during the period under study and, even more concerning, 43% of the files contained only one creatinine measure or no reference to this information, at all. 

These results become more of a problem if we consider that both Brazilian and international guidelines recommend annual measurement of creatinine in hypertensive and diabetic patients and the use of GFR to screen for CKD^12^ ^-^ ^13^ ^,^ ^16^.

Failure to comply with these guidelines when monitoring CKD is not limited to the state of Minas Gerais. Other authors list severe deficiencies in the management of renal disease within the PHC sphere. A study conducted in the south of Brazil reports that only 8% of diabetic patients and 5% of hypertensive patients had their GFR assessed by physicians in the PHC service^17^. In the state of São Paulo, records of microalbuminuria were found in only 1.4% of the medical files^18^. 

Another study conducted in the United States reports that 64% of PHC physicians report a lack of knowledge concerning criteria used to refer patients to specialized care services and 16% were not able to estimate a patient's glomerular filtration rate. The same study shows that 53% of the nephrologists interviewed believed that PHC physicians were late in referring patients to specialized assessment^3^.

Late referral of patients with CKD implies increased risk of mortality, worse metabolic status in dialysis, complications related to the use of temporary vascular access, longer hospitalizations, more difficult access to preemptive kidney transplantation, and a consequent increase in health services costs^5^ ^,^ ^19^. 

A retrospective study conducted in a dialysis center revealed that 22% of the patients were referred to secondary care services when in stage 5 of CKD (GFR< 15mL/min/1.73 m²), which resulted in a lower number of patients having a permanent vascular access to initiate dialysis^20^. Late referral was also observed in England, where approximately one quarter of the patients were referred to a specialist only one month before the need to initiate renal replacement therapy. Late referral was associated with a lower prevalence of preventive interventions, worse clinical status at the beginning of renal replacement therapy, longer hospitalizations, and lower survival rates^21^.

On the other hand, early referral of patients with CKD to the secondary care level is associated with a better prognosis. A recent study addressing 3,273 chronic patients at stages 3 to 5 verified that those referred to a nephrologist at advanced stages of the disease were at a greater risk of death before dialysis was initiated^22^. In addition to early referral of patients to a specialist, a multidisciplinary approach to renal disease can lead to more satisfying outcomes. A recent study reports that the average annual decline in the glomerular filtration rate was twice as high among patients monitored by a nephrologist only compared to when the treatment was conducted by a multidisciplinary team^23^. Similar results were obtained in the Hiperdia Minas Center in Juiz de Fora from 934 patients received cared over a period of two years. The speed of annual GFR loss in this sample was reduced or stabilized in two thirds of the patients, suggesting the multidisciplinary team was efficient in managing CKD^24^.

Favorable outcomes related to the progression of renal function among patients monitored in PHC units classified as "capable", allied with greater knowledge of early referral criteria, indicate the need to improve the structure and to systematize the work processes of those units classified as partially or not capable in order to harmonize the actions directed to patients with CKD among the various levels of healthcare services.

Even though a single city was addressed in this study, which can be considered one of this study's limitation, the results can support the sensitization of managers concerning the need to invest in the qualification of professionals and in management tools, coupled with a care plan that is shared between PHC and a multidisciplinary team, factors that can positively impact the care provided to individuals with CKD^25^. 

It is believed that the care provided to individuals with CKD in most Brazilian cities is even worse, considering there is a lack of programs connecting the services and harmonizing the care provided for this health condition.

## Conclusion

This study showed that the PHC units with higher scores concerning structure and process presented better clinical outcomes regarding the care provided to CKD, characterized by earlier referrals and lower declines in patient GFR rates. 

These findings suggest that the healthcare network is more efficient in providing care to chronic renal patients when PHC services develop appropriate clinical management processes, characterized by preventive, management and treatment actions directed to CKD.
